# Blastocyst Injection of Wild Type Embryonic Stem Cells Induces Global Corrections in Mdx Mice

**DOI:** 10.1371/journal.pone.0004759

**Published:** 2009-03-11

**Authors:** Elizabeth Stillwell, Joseph Vitale, Qingshi Zhao, Amanda Beck, Joel Schneider, Farah Khadim, Genie Elson, Aneela Altaf, Ghassan Yehia, Jia-hui Dong, Jing Liu, Willie Mark, Mantu Bhaumik, Robert Grange, Diego Fraidenraich

**Affiliations:** 1 Department of Cell Biology and Molecular Medicine, University of Medicine and Dentistry of New Jersey, New Jersey Medical School, Newark, New Jersey, United States of America; 2 Developmental Biology Program, Memorial Sloan-Kettering Cancer Center, New York, New York, United States of America; 3 Department of Pediatrics, University of Medicine and Dentistry of New Jersey, Robert Wood Johnson Medical School, Piscataway, New Jersey, United States of America; 4 Department of Human Nutrition, Foods and Exercise, Virginia Polytechnic Institute and State University, Blacksburg, Virginia, United States of America; Istituto Dermopatico dell'Immacolata, Italy

## Abstract

Duchenne muscular dystrophy (DMD) is an incurable neuromuscular degenerative disease, caused by a mutation in the dystrophin gene. Mdx mice recapitulate DMD features. Here we show that injection of wild-type (WT) embryonic stem cells (ESCs) into mdx blastocysts produces mice with improved pathology and function. A small fraction of WT ESCs incorporates into the mdx mouse nonuniformly to upregulate protein levels of dystrophin in the skeletal muscle. The chimeric muscle shows reduced regeneration and restores dystrobrevin, a dystrophin-related protein, in areas with high and with low dystrophin content. WT ESC injection increases the amount of fat in the chimeras to reach WT levels. ESC injection without dystrophin does not prevent the appearance of phenotypes in the skeletal muscle or in the fat. Thus, dystrophin supplied by the ESCs reverses disease in mdx mice globally in a dose-dependent manner.

## Introduction

DMD is an X-linked recessive disorder affecting 1 in 3500 newborn males. DMD is characterized by progressive muscle weakness associated with necrosis of muscle fibers and fibrosis of muscle tissue. Patients are usually confined to wheelchairs by age 8–11 years and generally die of respiratory failure by their early twenties [1], [2]. Although several therapeutic venues have been investigated [3]–[14], there is currently no cure.

DMD is caused by defects in the dystrophin gene. The full isoform (427 kD) of dystrophin localizes to the sarcolemma of the skeletal muscle. Dystrophin forms a complex with other proteins including dystroglycans, sarcoglycans, dystrobrevin and syntrophin called the dystrophin-glycoprotein complex (DGC) [15]. The DGC provides stability to the membrane and protects the skeletal muscle from mechanically induced damage. The C terminus of dystrophin is dispensable, partly because dystrobrevin (a distant dystrophin-related protein) can substitute for some of the functions of dystrophin, like binding to syntrophin. Syntrophin is responsible for localizing neuronal nitric oxide synthase (nNOS) to the sarcolemma. In the absence of dystrophin, the DGC is unstable and is destroyed [16]–[21].

Research on DMD has benefited from the availability of mdx mice, in which a naturally occurring mutation in exon 23 of the dystrophin gene abrogates expression of the full-length dystrophin polypeptide, causing symptoms reminiscent of muscular dystrophy [22], [23]. These mice exhibit moderate signs of skeletal muscle dystrophy but have nearly normal lifespan. This mild phenotype is due in part to compensatory upregulation of the dystrophin-related protein utrophin [24]–[26]. Previous studies in mice showed that only a fraction of the muscle must express full-length dystrophin to confer protection against the development of muscular dystrophy, as mice with a mosaic pattern of expression in the skeletal muscle displayed a markedly milder phenotype than mdx mice, despite the expression of dystrophin in only half the muscle fibers [27].

In addition to skeletal muscle, the full isoform of dystrophin is expressed in the cardiac and smooth muscles, and the brain [28], [29]. A short C-terminal isoform of dystrophin (apodystrophin, Dp71 or non-muscle dystrophin) is widely distributed in various non-muscle tissues, but Dp71 is not affected in mdx and its absence does not contribute to muscular dystrophy [24], [30]. Although the primary defect in the skeletal muscle is very well characterized, the relationship between the absence of the protein dystrophin and the pathogenetic mechanisms of DMD is still unclear. For example, abnormal vasoregulation in the exercising muscles of DMD patients and in the mdx muscle contributes to the vascular and dystrophic phenotypes, and forced expression of dystrophin in the smooth muscle of mdx mice partially corrects the phenotype [31], [32]. Thus, it appears that nonskeletal muscle full-length dystrophin plays a corrective role in the disease. This observation led us to hypothesize that partial restoration of dystrophin in all tissues could uncover novel corrective effects of dystrophin.

To study global corrections, we injected wild type ESCs into mdx blastocysts. We created chimeric mice composed by dystrophin positive ES-derived cells and dystrophin-negative mdx cells. ESCs incorporated at a low percentage but globally throughout the chimeric mouse. We report that dystrophin supplied by WT ESCs not only stabilizes the skeletal muscle in areas with high dystrophin and in areas with low dystrophin, but also corrects tissues that are affected in mdx and in DMD outside of the muscle, such as fat.

## Results

We wanted to determine if early injection of ESCs could revert phenotypes in mdx mice globally. To this end, we injected WT ROSA26 (R26) LacZ-marked ESCs (WT ESCs) into mdx blastocysts and examined the resultant mice (WT/mdx chimeras) at 4 months of age. At 3 weeks of age, the degree of chimerism was estimated by performing Xgal staining from sections of tail biopsies. After sacrifice, the degree of chimerism was assessed by Xgal staining in heart, liver and lung sections. The degree of chimerism was confirmed by dystrophin staining in heart sections and by dystrophin detection in western blots with protein extracts from hearts. Because skeletal muscle is syncytialized, neither Xgal (β-galactosidase is sarcoplasmic) nor dystrophin staining reflects the degree of chimerism. Therefore, the degree of chimerism in skeletal muscle was assessed by semi-quantitative PCR detection of the LacZ transgene in the R26 locus. Mdx heterozygous females contain one functional allele (50%) and are non-dystrophic [33]. Thus, we selected WT/mdx chimeras having less than 30% of WT ESC incorporation (n = 9 in the range of 10–30%, n = 2 in the range of 5–10% and n = 12 in the range of less than 5%).

### WT/mdx mice exhibit improved pathology and skeletal muscle function

**Figure 1:**
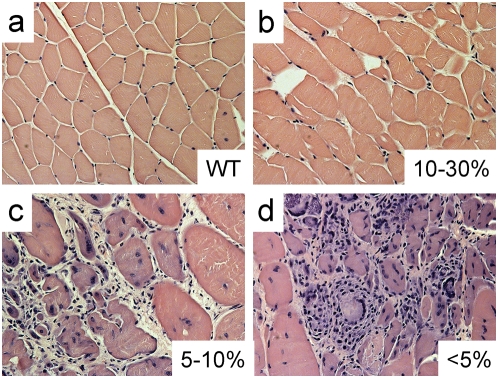
ESC incorporation prevents mononuclear invasion and central nucleation. a–d, Tibialis anterior (TA) muscle from WT (a), 10–30% WT/mdx (b), 5–10% WT/mdx (c) and <5% WT/mdx (d) mice were paraffin sectioned and H&E stained. Magnification of a–d: 200×. More than 2 animals per group (WT [n = 5], 10–30% WT/mdx [n = 5], 5–10% WT/mdx [n = 2] and <5% WT/mdx [n = 5]) were analyzed.

**Figure 2:**
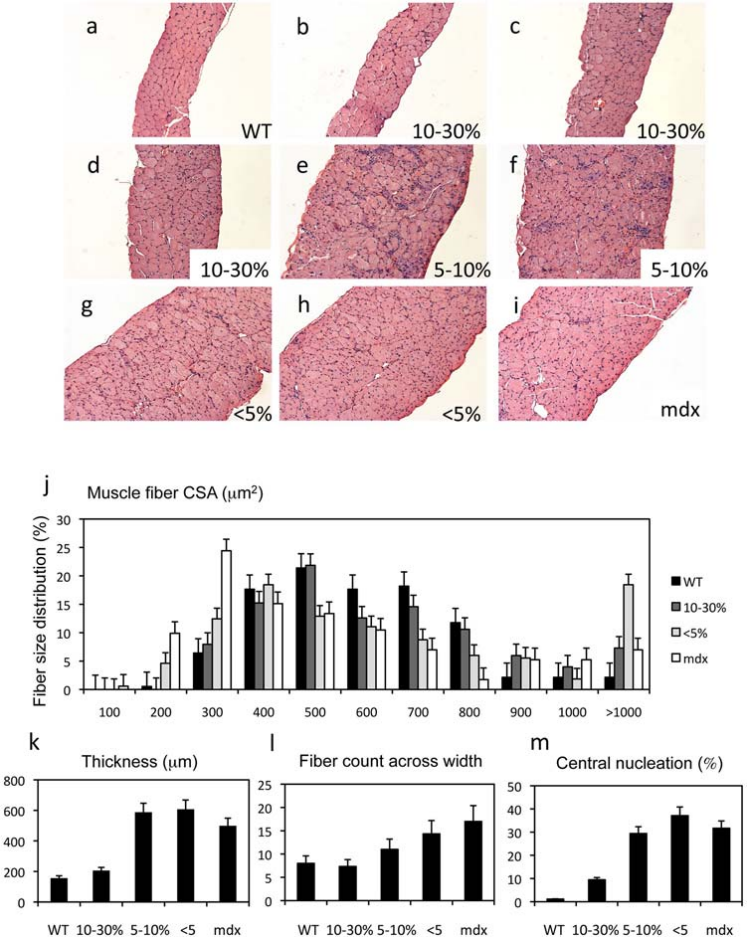
WT/mdx chimeric muscle exhibits improved pathology in a dose-dependent manner. a–i, Diaphragms from 4 month-old WT (a), 10–30% WT/mdx chimeric (b–d), 5–10% WT/mdx chimeric (e–f), less than 5% chimeric WT/mdx (g, h) and an mdx (i) mice were paraffin sectioned and H&E stained. j–m, fiber size distribution (j), thickness (k), fiber count across the diaphragm width (l) and percentage of central nucleation (m) measurements. Note that the thickness, the number of fibers across the diaphragm width or the percentage of central nucleation depends on the percentage of ESCs incorporated. Two or three diaphragm muscles per animal per group (WT [n = 3], mdx [n = 3], 10–30% WT/mdx [n = 3], 5–10% WT/mdx [n = 2] and <5% WT/mdx [n = 3]) were analyzed. At least 200 fibers were analyzed per animal per group for the studies indicated in (j) and (m). 20 measurements of thickness, with the corresponding number of fibers, per animal per group were performed in the studies indicated in (k) and (m). Magnification of a–i: 100×.

Gross examination indicated that, unlike mdx mice, hindlimb muscles from WT ESC-injected mice (10–30% incorporation) did not appear enlarged (data not shown). When suspended by the tail, chimeric mice straightened their hindlimbs. Histological analysis of muscles (pectoralis, quadriceps, tibialis anterior [TA]) from 10–30% WT/mdx mice displayed not more than 30% of central nucleation (in contrast to 60–90% of central nucleation in mdx or chimeric muscle with fewer than 5% of ESC incorporation)(Fig 1 and Table 1). In addition, the appearance of necrotic areas, extensive mononuclear invasion and lack of an organized architecture in the 5–10% and <5% chimeric muscle (Fig 1c and d) and in the mdx muscle (data not shown) was reversed in the 10–30% chimeric muscle (Fig 1b, compare to Fig 1a, Table 1). Unlike mdx and <5% chimeric diaphragms, which were thicker (450–650 µm), contained 12–24 fibers across the width and displayed greater than 20% of central nucleation, WT or 10–30% chimeric diaphragms showed no such abnormalities (thickness: 160–250 µm, 5–10 fibers across the width, less than 10% of central nucleation) (P<0.001)(Fig. 2a–i, k–m, Table 1). The fiber cross-sectional area (CSA) distribution of WT and 10–30% chimeric diaphragms co-peaked at 500 µm^2^ (mode) and the values were higher than those of the mdx or <5% chimeric diaphragms (300 µm^2^ and 400 µm^2^ respectively), probably due to the presence of small caliber, regenerating (<300 µm^2^) fibers in the mdx or <5% muscles (Fig. 2j and Table 1). Interestingly, all the chimeric and the mdx diaphragms exhibited a high percentage of fibers with CSAs greater than 900 µm^2^ (26% for 10–30% chimeras, 56%<5% chimeras and 17% for mdx compared to 6% for WT)(Fig. 2j), suggesting the presence of hypertrophic fibers in the chimeric muscles (P<0.05). Indeed, some fibers of <5% chimeras exceeded CSAs of 3,000 µm^2^. Consistent with histological corrections, the extensor digitorum longus (EDL) from 10–30%, but not from <5% WT/mdx chimeras exhibited functional improvement. Stress output (force/cross sectional area) for tetanic contractions (42.8±3.6 g/mm^2^, n = 4) and twitch contractions (7.0±0.8 g/mm^2^, n = 4) from the EDL of 10–30% chimeras but not from <5% chimeras (22.2±2.2 tetanic: g/mm^2^; twitch: 4.8±0.4 g/mm^2^, n = 4) were comparable to that of WT mice (tetanic: 46.4±2.5; twitch: 6.9±0.7 g/mm^2^; n = 4), and significantly higher from that of mdx mice (tetanic: 20.0±7.9; twitch: 5.2±2.0 g/mm^2^, n = 3). (P<0.05 between 10–30% [or WT] versus <5% chimeras [or mdx])(Table 1). Thus, incorporation of WT ESCs into mdx mice improves skeletal muscle pathology and function in a dose-dependent manner.

**Table 1:** The degree of chimerism correlates with the degree of corrections.

	WT	chim10–30	chim5–10	chim<5	mdx
Xgal staining (% of cells or skeletal muscle fibers)					
heart	0	10–30	<5	<5	0
liver, lung, brain	0	5–30	<5	UD	0
quadriceps	0	50–70	<5	UD	0
pectoralis	0	50–90	10–30	<5	0
diaphragm	0	20–50	UD	UD	0
Dystrophin (WB, % relative to WT)					
heart	100	10–30	5–10	<5	0
quadriceps	100	60–100	<5	0	0
pectoralis	100	70–100	5–10	0	0
diaphragm	100	30–60	10–15	<5	0
Fiber cross-sectional area (µm^2^)					
diaphragm (mode)	500	500	400	400	300
Central nucleation (%)					
quadriceps, TA, pectoralis	<10	10–30	ND	60–90	60–90
diaphragm	<5	<10	20–40	25–45	20–40
Muscle thickness (µm)					
diaphragm	160–180	180–250	540–600	550–650	450–550
Fiber count across width					
diaphragm	6–10	5–10	8–13	12–18	18–24
Mononuclear invasion*					
dorsal diaphragm, pectoralis, TA	−	++	+++	++++++	++++++
Dystrophin glycoprotein complex (IMF)(dbv-α, synt, nNOS, SG-β)					
diaphragm	++++++	++++++	+++	++	++
Sarcolemmal utrophin (IMF)					
diaphragm	−	++	ND	++++++	++++++
eMHC (IMF, fold increase by microarrays)					
diaphragm, pectoralis	−	++, UC	++++, ND	++++++, ND	++++++, 24
Mechanical stress (g/mm^2^)					
EDL (tetanus)	46.4±2.5**	42.8±3.6	ND	22.2±2.2	20.0±7.9**
EDL (twitch)	6.9±0.7**	7.0±0.8	ND	4.8±0.4	5.2±2.0**

chim10–30: chimeric mice that incorporated between 10 and 30% of ESCs (n = 9); chim5–10 (n = 2); chim<5 (n = 12); WT (n>10); mdx (n>10).

UC: unchanged; UD: undetectable; ND: not determined; IMF: immunofluorescence.

dbv-α: dystrobrevin-α; syntrophin: synt; neuronal nitric oxide synthase: nNOS; sarcoglycan β: SG-β.

* ++low degree; ++++++ high degree.

** WT and mdx twitch and tetanic stress values are unpublished data from A.Wolff's dissertation (Virginia Polytechnic Institute and State University, 2007).

### R26 ESC-derived tissues produce β-galactosidase

**Figure 3:**
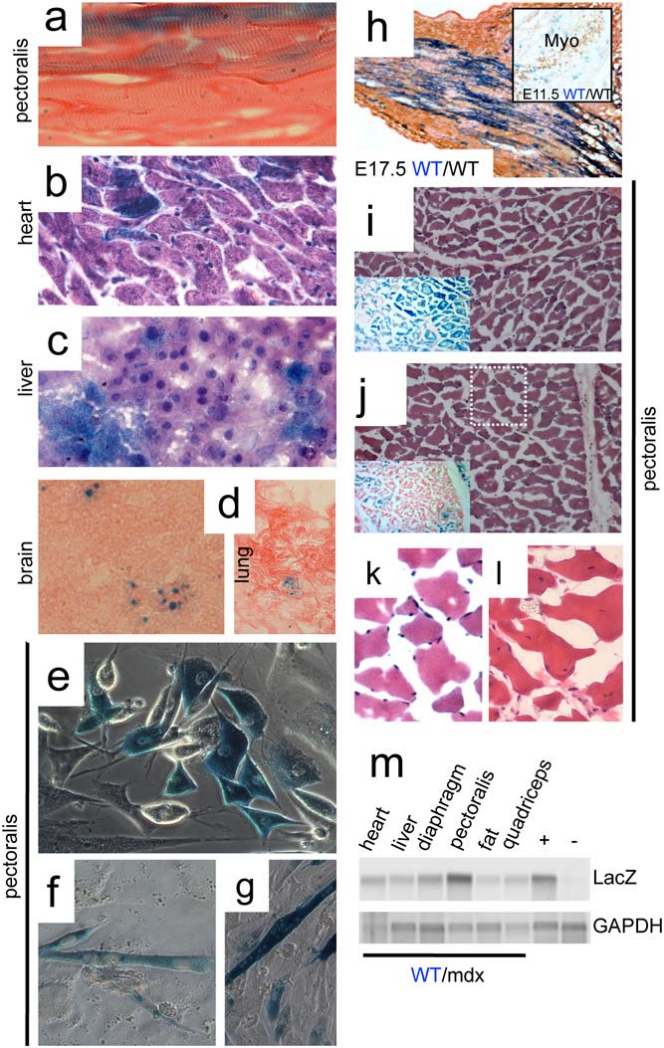
β-galactosidase activity is detected in chimeric tissues. a–d, Xgal (blue) staining of sections of pectoralis muscle (a, longitudinal), heart (b), liver (c), brain (d, left) and lung (d, right) from 10–30% R26 WT/mdx chimeric mice (400× for a–c and 200× for d). a and d: eosin counterstained; b and c: H&E counterstained. Xgal staining in (d) (brain and lung) was weak and punctuated. e–g, myogenic cells (e) were isolated from pectoralis muscle of 10–30% R26 WT/mdx chimeric mice, and subjected to differentiation into multinucleated, contracting myotubes (f, g)(400×). h, E17.5 R26 WT/WT embryos were collected, cryosectioned at the hindlimb level and Xgal/eosin stained (200×). Adjacent sections were MHC (MF20) immunostained to visualize the developing muscle (not shown). h inset, E11.5 R26 WT/WT embryos were collected, whole mount Xgal stained, cryosectioned at the thoracic level and myogenin (Myo) immunostained to visualize the myotomes (200×). i, j, insets, Xgal/eosin staining of pectoralis muscle (transverse) cryosections from 10–30% R26 WT/mdx chimeric mice displaying uniform Xgal (i, inset) and non-uniform Xgal (j, inset) staining (100×). i, j, H&E staining of areas adjacent to i, j, insets (100×). k is a magnification (400×) of the dotted white box in (j) showing negligible Xgal staining. l, pectoralis muscles from mdx mice were cryosectioned and H&E stained. Note that while (k) shows peripheral nucleation, (l) shows central nucleation. m, genomic DNA was isolated from various tissues of 10–30% R26 WT/mdx chimeras and subjected to PCR for β-galactosidase. +: positive control with DNA from the tail of a R26 WT mouse. −: negative control with DNA from the tail of a non-R26 WT mouse. Xgal staining experiments were performed with muscles, heart, liver, lung and brain tissues of all WT/mdx chimeras (n = 9 for 10–30%, n = 2 for 5–10% and n = 12 for <5% WT/mdx) and percentage of positive fibers are indicated in Table I. Myogenic cells were derived from pectoralis and quadriceps muscles of two 10–30% chimeras (n = 2). LacZ PCR was performed with DNA from two 10–30% chimeras (n = 2). Five WT/WT embryos at E11.5 (n = 5) and five WT/WT embryos at E17.5 (n = 5) were Xgal stained.

As expected for a syncytium, the muscle from 10–30% chimeras contained 50–90% of X-gal stained fibers (Fig. 3 and Table 1), while other tissues of the same chimeras (liver, heart, brain and lung) contained less than 30% of X-gal stained cells (Fig. 3b–d and Table 1) (P<0.001). The diaphragm contained more blue fibers (20–50% of blue fibers, Table 1) than blue cells from the liver (10–30% of blue cells, Fig. 3c and Table 1), but less than the quadriceps, pectoralis or TA (Fig. 3a, i, j, Table 1 and data not shown). Genomic PCR confirmed the presence of the LacZ allele in all tissues analyzed (Fig. 3m), indicating widespread incorporation of ESCs into the mouse chimera. The pattern of X-gal staining in muscle was non-uniform, with varying intensities. Blue staining varied from fiber to fiber, and also from different portions within the same fibers (Fig. 3a and 3i, j, insets). Importantly, muscle areas with minimal X-gal staining, but surrounded by areas with high number of blue fibers (Fig. 3j, inset) displayed peripheral nucleation (Fig. 3k [magnification of white dotted box in 3j]), indicating no regenerating activity. As expected, non-blue fibers were more abundant in an animal with 10% than in another with 30% of WT R26 ESCs, with concomitant appearance of a higher percentage of central nucleation and mononuclear invasion (data not shown).

Because the skeletal muscle regenerates via activation of myogenic precursor cells, we wanted to know if these cells were also derived from ESCs. A fraction of the myogenic cells derived from chimeric muscle was also Xgal positive (Fig. 3e), and contracting multinucleated myotubes produced from this preparation were also blue (Fig. 3f and g). Because the skeletal muscle of the WT/mdx chimeras displayed a large percentage of Xgal positive fibers distributed throughout the muscle (spatial expansion), we wanted to know if the spatial expansion of the R26 ESCs in the muscle required the presence of an mdx component. We also wanted to determine if this effect appeared during development or postnatally. To this end, we injected R26 ESCs into WT blastocysts, and generated WT/WT chimeras. We stained the embryos with Xgal and examined muscle precursors at developmental stages E11.5 and at E17.5 (Fig. 3h, inset and 3h). No preferential staining was observed in the myotomes at E11.5 (where no multinucleation is apparent). However, spatial expansion was observed in the muscle of the E17.5 embryos (where multinucleation becomes apparent). Thus, expansion of Xgal staining throughout the muscle occurs at late stages of development, and does not require mdx blastocysts as recipient embryos.

**Figure 4:**
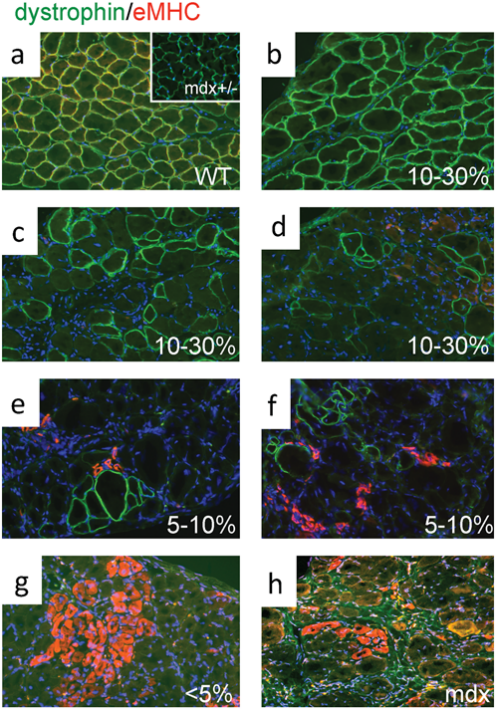
Embryonic MHC (eMHC) is greatly reduced in the skeletal muscle of R26 WT/mdx chimeras. a–d, diaphragms from WT (a), mdx+/− (a, inset), 10–30% WT/mdx (b–d), 5–10% WT/mdx (e, f), <5% WT/mdx (g) and mdx (h) mice were sectioned and stained for dystrophin (green) and eMHC (red). b–d, areas with high (b) and low (c, d) numbers of dystrophin-positive fibers within the same WT/mdx diaphragm. e, f, all areas within the 5–10% WT/mdx diaphragm display low numbers of dystrophin-positive fibers. DAPI (blue) demarcates nuclei. Magnification: 200×. eMHC detection experiments were performed with pectoralis, diaphragm and quadriceps muscles of 2–4 animals per group (WT [n = 4], mdx+/− [n = 2], 10–30% WT/mdx [n = 4], 5–10% WT/mdx [n = 2], <5% WT/mdx [n = 4] and mdx [n = 3]).

**Figure 5:**
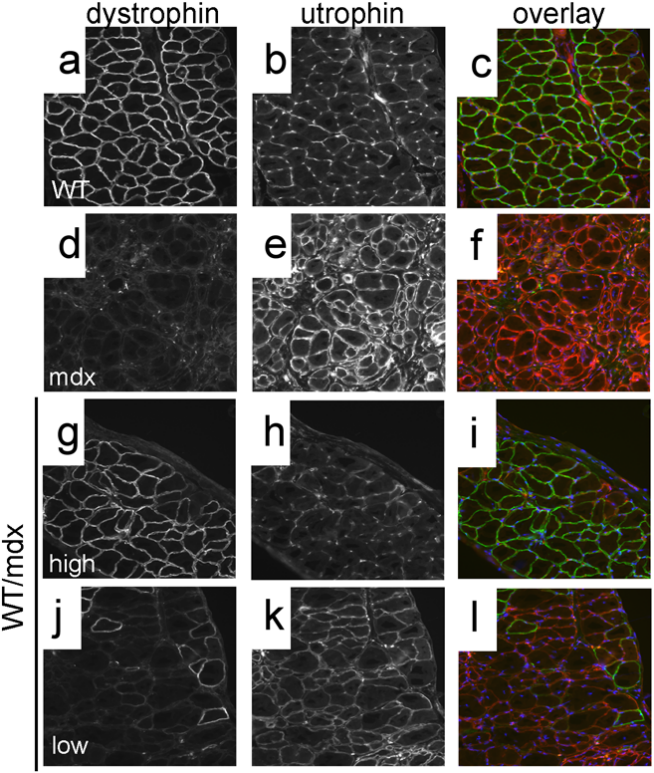
WT/mdx chimeric muscle is composed of dystrophin-positive (utrophin-negative) and dystrophin-negative (utrophin-positive) fibers. a–l, diaphragms from WT (a–c), mdx (d–f) and 10–30% WT/mdx chimeric (g–l) mice were sectioned and stained for dystrophin (a,d,g,j) and utrophin (b,e,h,k). (c,f,i,l): dystrophin (green)/utrophin (red) overlay. Note absence of yellow (overlay of green and red), indicating no dystrophin/utrophin co-localization. g–i, areas of high dystrophin; j–l, areas of low dystrophin in the diaphragm of the same mouse. Magnification: 200×. Experiments were performed with pectoralis and diaphragm muscles of 3 animals (n = 3) per group (WT, 10–30% WT/mdx and mdx).

**Figure 6:**
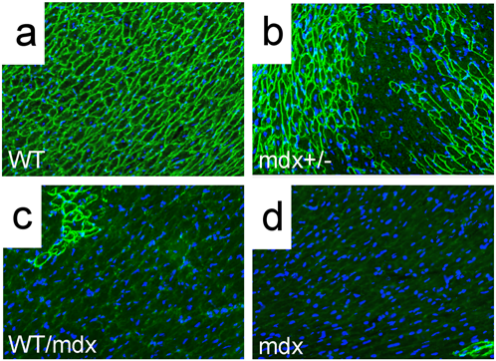
Dystrophin is detected in cardiomyocytes in the WT/mdx chimeric heart. Dystrophin immunofluorescence (green) was performed on sections of WT (a), mdx+/− (b), 10–30% WT/mdx chimeric (c) and mdx (d) heart (400×). Note the presence of revertant cardiomyocytes in (d, bottom right). DAPI (blue) demarcates nuclei. Experiments were performed with 3 animals (n = 3) per group (WT, mdx+/−, 10–30% WT/mdx and mdx).

Immunofluorescence analysis using a dystrophin antibody confirmed the observations obtained with X-gal staining: 50–90% (pectoralis, quadriceps and TA) and 30–70% (diaphragm) of the 10–30% WT/mdx skeletal fibers were positive for dystrophin (with varying intensities)(Fig 4b–d and Fig. 5g, j; compare to Fig. 5a, d). This is in contrast to the low numbers of dystrophin-positive cardiomyocytes (10–30%) (Fig. 6c, compare to Fig. 6a, b)(P<0.001) observed in the same 10–30% chimeric animals. Dystrophin-negative fibers as well as dystrophin-negative cardiomyocytes were positive for the related protein utrophin (Fig. 5j–l and data not shown for cardiomyocytes).

### WT/mdx muscle overproduces dystrophin

**Figure 7:**
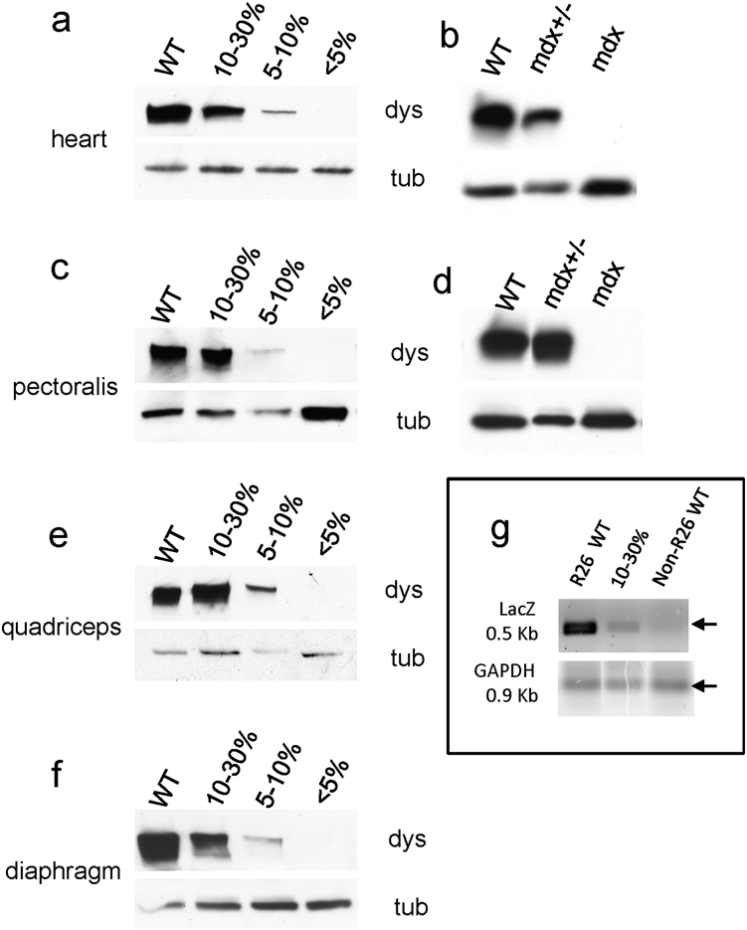
WT/mdx chimeric muscle overproduces dystrophin. a, c, e and f, protein extracts from hearts (a), pectoralis (c), quadriceps (e) and diaphragms (f) of WT, 10–30% WT/mdx, 5–10% WT/mdx and <5% WT/mdx mice were subjected to a western blot for dystrophin and tubulin (load control). Band intensities (dystrophin/tubulin) were scanned and values relative to WT (100%) are presented in Table I. b, d, protein extracts from hearts (b) or pectoralis muscles (d) of WT, mdx+/− and mdx mice were subjected to a western blot for dystrophin and tubulin. Western blots were performed with extracts of hearts and various muscles (pectoralis, quadriceps and diaphragm) from 2–4 animals per group (WT [n = 3], mdx+/− [n = 2], 10–30% [n = 4], 5–10% [n = 2], <5% [n = 4] and mdx [n = 3]). g, DNA from 10–30% (n = 4) R26 WT/mdx, R26 WT (n = 2) and non-R26 WT (n = 3) mice were isolated from quadriceps muscles and subjected to semi-quantitative PCR using primers for LacZ or GAPDH. Note in that the 10–30% quadriceps muscle shows normal levels of dystrophin (relative to WT) (e) but only a fraction of LacZ-positive DNA (relative to R26 WT) (g).

We determined the degree of ESC incorporation in the WT/mdx skeletal muscles by quantitating the percentage of DNA containing the LacZ transgene. To this end, we isolated DNA from skeletal muscle (and liver) of R26/mdx chimeras. We also isolated DNA from skeletal muscle (and liver) of R26 mice, which contain the LacZ transgene in all the nuclei (and from whom the R26 ESCs were derived), as well as DNA from skeletal muscle (and liver) of WT and of mdx mice, which contain no LacZ transgene. In skeletal muscle, most of the DNA comes from the myonuclei. We performed semi-quantitative PCR with primers that specifically amplify the LacZ transgene from R26 ESC-derived but not from the mdx nuclei in the chimera. As expected, the degree of chimerism in the liver ([chimeric liver/R26 liver]×100) of the chimeric animals was low (5–15%), and roughly overlaps with the value of β-gal positive cells, which is also low (5–30%, Table 1). The degree of chimerism in the muscle ([chimeric muscle/R26 muscle]×100) of the 10–30% chimeric animals was also low at 11–19% (Fig 7g), but did not overlap with the high percentage of β-gal positive fibers (50–70% in the quadriceps, 50–90% in the pectoralis or 20–50% in the diaphragm, Table 1). This indicates that even though a large percentage of the muscle fibers contain β-gal activity, there is no preferential homing of R26 ESCs to the skeletal muscle compared to other tissues such as lung, liver or heart. Thus, all tissues within a WT/mdx animal contain a similar (low) degree of mosaicism.

To determine if dystrophin protein levels in the chimeric muscle correlated to the degree of chimerism, we performed western blots on muscle tissue (pectoralis, diaphragm and quadriceps) from chimeric mice (Fig. 7). Values relative to dystrophin protein present in the WT muscle (100%) were obtained by band scanning and normalized by tubulin according to the formula: dystrophin chimera/tubulin chimera]/[dystrophin WT/tubulin WT]×100 (Table 1). As expected, in the heart of the 10–30% WT/mdx chimeras, where no syncytia form, dystrophin levels reached 10–30% of that seen in WT mice (Table 1 and Fig. 5a). However, in the quadriceps and pectoralis (and at a lower extent in the diaphragm) where syncytia develop, dystrophin levels reached 70–100% (pectoralis), 60–100% (quadriceps) and 30–60% (diaphragm) of that seen in WT mice (Table 1, Fig. 7c, e and f for pectoralis, quadriceps and diaphragm respectively). The observation that dystrophin levels are elevated in skeletal muscle relative to heart muscle with comparable extent of chimerism suggests that WT ESC-derived myonuclei have the ability to overproduce dystrophin to compensate for the inability of the mdx myonuclei to produce the protein. A compensatory effect was also observed in the mdx+/− mice (mdx heterozygous mice recapitulate DMD asymptomatic carriers). Because of X-chromosome inactivation, only half of the cardiomyocytes contain dystrophin (Fig. 6b, compare to Fig. 6a), whereas all skeletal muscle fibers contain dystrophin (Fig. 4a, inset). Concordant with this finding, 50% of the WT level of dystrophin was observed in the heart of the mdx heterozygous mice, but 100% of WT levels were observed in the skeletal muscle (Fig. 7b, d). Thus, overproduction of dystrophin occurs in the skeletal muscle but not in the heart. In addition, since the mdx+/− mice have not received ESCs, overproduction of dystrophin in a chimeric muscle does not require ESC injection.

Using microarray analysis we confirmed previous reports [34] showing that dystrophin RNA in the skeletal muscle of the mdx mouse is reduced (Tables 2 and S1), probably because most of the mutated transcript is inactive and thus destroyed. However, dystrophin RNA levels in the WT/mdx mice were unchanged relative to WT mice (Tables 2 and S2), even though WT/mdx mice contained only 10–30% of WT cells. Thus, elevation of dystrophin RNA accompanies elevation of dystrophin protein.

**Table 2:** Markers of muscular dystrophy are reversed in WT/mdx but not in mdx/mdx muscle.

name	accession number	fold change		
		mdx vs WT	WT/mdx vs WT	mdx/mdx vs WT
dystrophin, DMD	1448665_at	−7.46	unchanged	−2.3
myosin, heavy polypeptide				
3, embryonic	1427115_at	24.25	unchanged	147
thymosin β10	1436902_x_at	4.29	unchanged	4.29
procollagen type I, α2	1423110_at	6.06	2.14	4.92
procollagen type III, α1	1427884_at	2	unchanged	5.28

### WT/mdx muscle shows reduced regeneration

**Figure 8:**
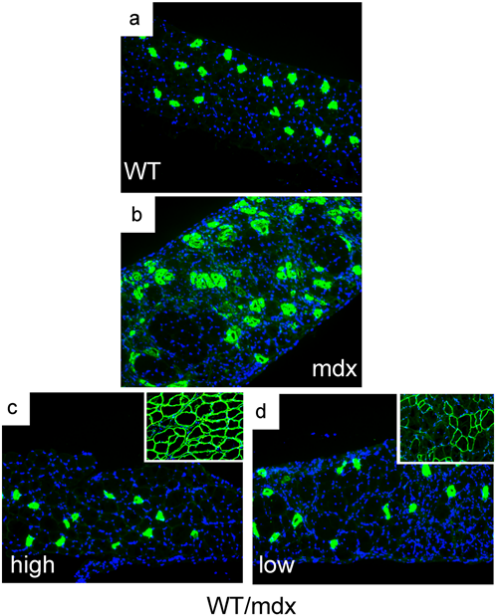
Slow MHC is not clustered in the skeletal muscle of WT/mdx chimeric mice. a–d, diaphragms from WT (a), mdx (b) and 10–30% WT/mdx chimeric (c, d) mice were sectioned and stained for slow MHC (green). c and d, insets, adjacent sections of (c) and (d) respectively from the same diaphragm stained for dystrophin. DAPI (blue) demarcates nuclei. Magnification: 200×. Experiments were performed with diaphragm muscles of 2 animals (n = 2) per group (WT, mdx and 10–30% WT/mdx).

**Figure 9:**
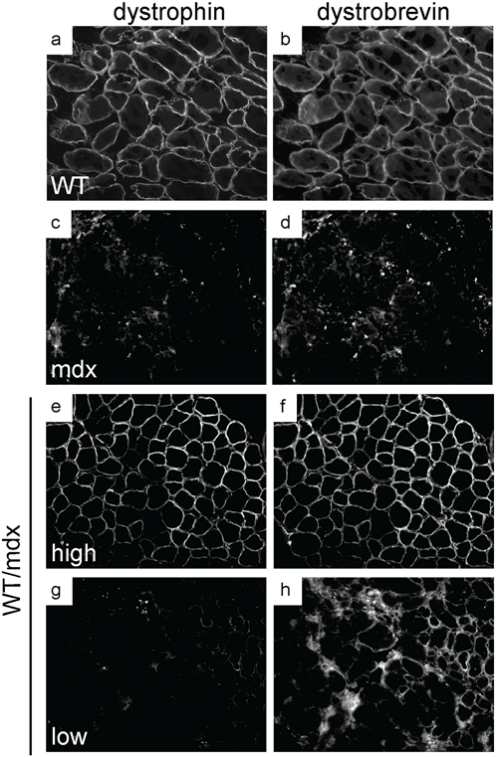
Dystrobrevin is present in areas with low dystrophin in WT/mdx muscle. a–h, diaphragms from WT (a, b), mdx (c, d), and 10–30% WT/mdx (e–h) were sectioned and stained for dystrophin (a, c, e, g) and dystrobrevin (b, d, f, h). e–h, areas with high dystrophin (e, f) and areas with low dystrophin (g, h) within the same muscle. Magnification: 200×. Experiments were performed with pectoralis and diaphragm muscles of 3 animals (n = 3) per group (WT, mdx and 10–30% WT/mdx).

**Figure 10:**
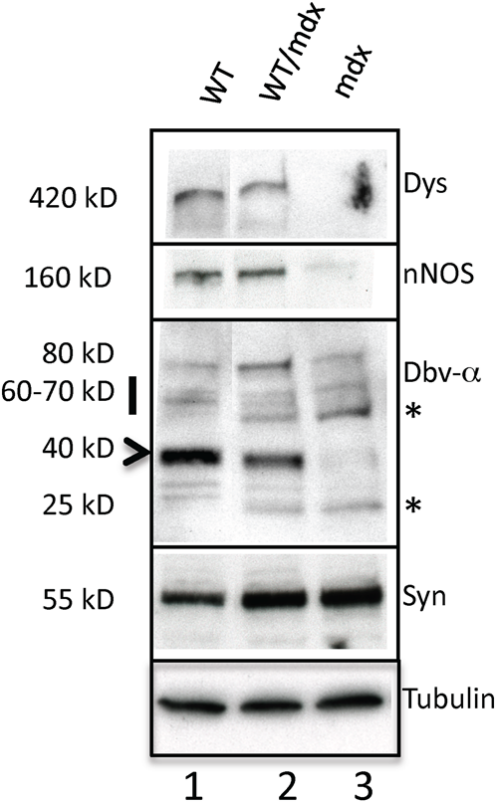
WT/mdx muscle displays nNOS and dystrobrevin-α. Protein extracts from diaphragms of WT (lane 1), 10–30% WT/mdx (lane 2) and mdx (lane 3) mice were subjected to a western blot for dystrophin, nNOS, dystrobrevin-α, syntrophin and tubulin. *: dystrobrevin-α isoforms enriched in mdx and WT/mdx but not in WT muscle. Experiments were performed with pectoralis, diaphragm and quadriceps muscles of 3 animals (n = 3) per group (WT, 10–30% WT/mdx and mdx).

**Figure 11:**
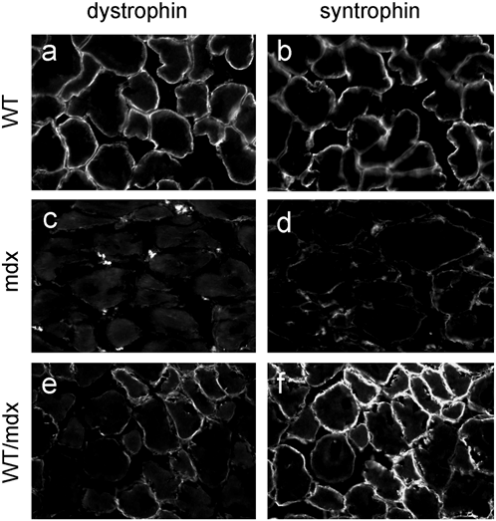
Syntrophin is present in WT/mdx but not in the mdx muscle. a–f, diaphragms from WT (a, b), mdx (c, d), and 10–30% WT/mdx (e–f) mice were sectioned and stained for dystrophin (a, c, e) and syntrophin (b, d, f). Magnification: 200×. Experiments were performed with diaphragms of 2 animals (n = 2) per group (WT, mdx and 10–30% WT/mdx).

To determine the molecular consequences of incorporation of WT ESCs into the mdx musculature, we performed microarray analysis to search for restoration of markers dysregulated in the mdx muscle. The embryonic isoform of myosin heavy chain (a marker of regeneration, eMHC, [35]), which was upregulated in the mdx muscle (Tables 2 and S1), was normalized in the 10–30% WT/mdx chimeric muscle (Tables 2 and S2). In addition, thymosin-β10 (a small actin-binding protein involved in cytoskeletal remodeling, [36]) and a number of procollagen genes (which are responsible for the formation of a fibrotic muscle, [37]), were also upregulated in the mdx muscle, and normalized in the 10–30% WT/mdx chimeric muscle (Tables 2, S1 and S2). An antibody reactive with eMHC did not detect areas of regenerating fibers in the WT or in 10–30% WT/mdx chimeras (Fig. 4a–d and Tables 1 and S2) even outside the areas of dystrophin-positive fibers (within the utrophin-positive areas) (Fig. 4c and d and Fig. 5j–l). By contrast, eMHC was detected in the 5–10% and the <5% WT/mdx as well as in the mdx muscle (Fig. 4e–h, Tables 1 and S1). This observation suggests that the dystrophin-negative (utrophin-positive) fibers of 10–30% WT/mdx muscle are stabilized, perhaps by the presence of the neighboring dystrophin-positive fibers or other dystrophin-positive tissues outside of the muscle. In support of the notion that correction of the skeletal muscle occurs as a whole, we found that while the slow isoform of MHC (sMHC) was grouped in clusters in the mdx diaphragm (Fig. 8b, compare to Fig. 8a), sMHC was restored to a normal pattern in the diaphragm of the 10–30% WT/mdx mice (Fig. 8c, d), in areas of high dystrophin (Fig. 8c, inset) and also in areas with low dystrophin (Fig. 8d, inset). Dystrobrevin-α localized to the sarcolemma of 10–30% WT/mdx muscle (Fig. 9e–h and Table 1), in areas with high dystrophin (Fig. 9e, f) and also in areas with low dystrophin (Fig. 9g, h). In western blots performed with 10–30% WT/mdx muscles (Fig. 10, lane 2), a major isoform of dystrobrevin-α of about 40 kD was detected at high levels. Other isoforms of dystrobrevin-α were detected. Variations in the molecular weight are apparent between some mdx and WT isoforms of dystrobrevin, with the chimeric samples containing both types (Fig. 10, lane 2, compare to lanes 1 and 3). The 40 kD isoform observed in the chimeras is an isoform observed predominantly in the WT sample (Fig. 10, lane 1). Thus, the levels of the 40 kD isoform of dystrobrevin correlate with the levels of full-length dystrophin. Similarly, nNOS was predominantly detected in the WT and in the chimeras (Fig. 10 and Table 1). Thus, nNOS levels correlate with dystrophin levels as well. Syntrophin levels however are not sensitive to dystrophin levels, since it was detected also in the western blot with an mdx sample (Fig. 10). However, immunodetection using the same antibody failed to localize syntrophin to the sarcolemma of the mdx muscle (Fig. 11 and Table 1). It is possible that in the absence of dystrophin, syntrophin fails to attach to the sarcolemma but is not destroyed.

### WT/mdx mice display corrections outside the skeletal muscle fibers

Within the muscle, corrections outside the skeletal fiber proper were also apparent. Myelin basic protein (MBP), a major component of the Schwann cells, was downregulated in the mdx muscle (Table S1: 4.29-fold decrease, mdx vs WT) whereas MBP levels in the 10–30% WT/mdx chimeric muscle were restored to normal (Table S2: unchanged, WT/mdx vs WT). Schwann processes, which guide the innervation of a newly regenerated fiber, are compromised in the mdx muscle [38]. Corrections outside the skeletal fibers were also apparent. Unlike mdx, which are lean, WT/mdx chimeric mice with 10–30% WT ESC chimerism had improved fat mass (abdominal fat weight/total weight of WTs: 4.2±1.5%, of mdx mice: 1.2±0.4%, of 10–30% WT/mdx chimeras: 4.0±1.4%) (P<0.05). The nearly 4-fold increase in the mass of the chimeric adipose tissue (relative to mdx adipose tissue) is proportional to the number of adipocytes, as both the mdx and the WT/mdx chimeric fat cells have comparable cell size (data not shown). To determine the molecular consequences of the incorporation of WT ESCs into the mdx fat, gene expression profiles were investigated. The mdx and the 10–30% WT/mdx chimeric fat displayed upregulation of several skeletal and cardiac muscle contractile genes (relative to WT fat)(Tables 3, S3 and S4). Among these markers were myosin, actin, tropomyosin and troponin isoforms (Table 3). Interestingly, follistatin-like, a secreted factor that binds and antagonizes the effect of myostatin and promotes muscle hypertrophy [39], was also upregulated in the mdx and the WT/mdx fat (13.93- and 27.86-fold increase relative to WT, Tables S3 and S4 respectively). The presence of follistatin-like 1 and 3, but not of follistatin, was confirmed in the 10–30% chimeric fat by RT-PCR (data not shown). Taken all together, these experiments suggest that WT ESCs injected into mdx blastocysts result in changes in non-muscle tissues like the fat. As a consequence of chimerism, the adipose tissue increases mass and upregulates muscle contractile genes and paracrine bioactive genes.

**Table 3:** Cardiac and skeletal muscle contractile markers are upregulated in mdx and WT/mdx adipose tissue.

name	accession number	fold change	
		mdx vs WT	WT/mdx vs WT
Actin, α1, skeletal muscle	1427768_s_at	UC	8.57
MyHC, fast skeletal muscle embryonic	1428655_at	−4.92	UC
MyHCα cardiac	1448826_at	147.03	UC
MyHC-IIX	1427520_a_at	UC	55.72
MyHC-IIB	1427026_at	16	48.5
MyH polypeptide 7, cardiac muscle β	1448554_s_at	16	UC
MyLC-alkali fast skeletal	1452651_a_at	UC	24.25
MyLC ventricular alkali	1427768_s_at	13.93	UC
MyLC phosphorylatable cardiac ventricles	1448394_at	4.59	4.29
Myosin X	1450650_at	4.59	4
Myosin 1B	1448989_a_at	UC	2.3
Myosin Vb	1452298_a_at	UC	2.14
Myosin Va	1431320_a_at	−2.83	UC
Tropomyosin-2β	1425028_a_at	3.73	17.15
Tropomyosin 3γ (non-muscle)	1427567_a_at	UC	−13
Tropomyosin-1α	1423049_a_at	17.15	25.99
Troponin C fast skeletal	1417464_at	32	97.01
Troponin T2 cardiac	1418726_a_at	36.76	24.25
Troponin T cardiac, isoform A2b	1424967_x_at	24.25	16
Troponin T3, skeletal fast	1450118_a_at	UC	6.5
Troponin C cardiac slow skeletal	1418370_at	6.5	3.73
Troponin I, cardiac	1422536_at	3.48	3.73

### Dystrophin from WT ESC-derived muscle is required to correct muscular dystrophy

**Figure 12:**
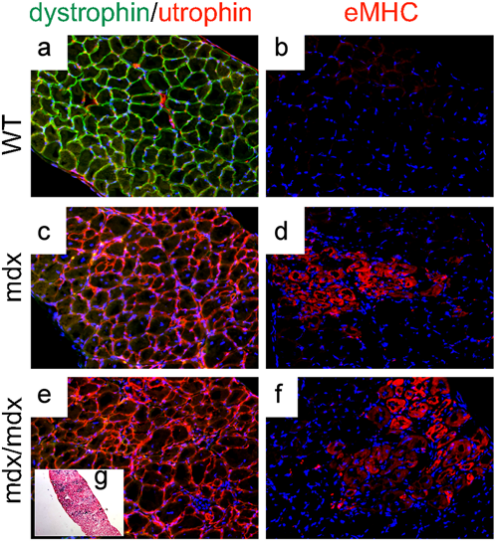
Mdx muscle receiving ESCs without dystrophin is dystrophic. a–f, diaphragms from WT (a, b), mdx (c, d) and R26 mdx/mdx chimeric (e, f) mice were sectioned and stained for dystrophin (green) and utrophin (red) (a, c, e) and for eMHC (b, d, f). DAPI (blue) demarcates nuclei (200×). g, Xgal/eosin staining of an adjacent section of (e, f) (100×). Experiments were performed with pectoralis, diaphragm and quadriceps muscles of 4 animals (n = 4) per group (WT, mdx and R26 mdx/mdx chimeras).

To determine if the rescue is dependent on the percentage of WT ESCs incorporated into the muscle syncytium also at the molecular level, we examined gene expression profiles with RNA from <5% chimeric muscle. Unlike the muscle from 10–30% chimeric mice (which showed reversion of dystrophic markers, Tables 2 and S2), microarray analysis from the <5% chimeric muscle showed no reversion (relative to WT: 6.5-fold decrease for dystrophin, 32-fold increase for embryonic myosin heavy polypeptide, 6.96-fold increase for thymosin-β10 and 4 procollagen genes increased). This suggests that the degree of molecular corrections depends on the number of WT ESCs incorporated and confirms our previous observations indicating that a minimal incorporation of ESCs (less than 5%) is insufficient to effect histological and functional corrections in the mdx musculature. To determine if dystrophin from the ESCs is required to effect corrections, we derived LacZ-marked ESC clones from mdx mice (Table 4). Two such mdx ESC clones with normal karyotypes (HE5 and HA4) were injected into mdx blastocysts to create chimeras with ESC-derived tissue without dystrophin (mdx/mdx chimeras, n = 7). A broad range of mdx/mdx chimeras examined (0–70% of mdx ESC incorporation) showed a dystrophic phenotype (Fig. 12e, f), with no correction of gene expression profiles (Tables 2 and S5). MBP failed to be normalized in the mdx/mdx muscle (Table S5: mdx/mdx vs WT: 2.0 fold-decrease for MBP). The fat mass phenotype also failed to be corrected by mdx ESC injection (abdominal fat weight/total weight: 0.9±0.2%, compare with WT/mdx chimeras: 4.0±1.4%, P<0.05). Thus, dystrophin from the ESCs is required to correct muscular dystrophy globally.

**Table 4:** LacZ, alkaline phosphatase and karyotype analysis of the mdx ESC clones.

Clone	LacZ	AP	Karyotype
HE5*	+	60% +	a predominantly normal male. A minor population lacks a Y chromosome.
A1-D4	+	90% +	The majority of cells have trisomy 12, male.
A2-B4	+	70% +	
GB3	+	90% +	predominantly normal female An elevated level of tetraploidy is present.
HG1	+	70% +	
HA4*	+	65% +	normal male
FA3	+	90% +	a mixture of cells of different origin.
A1-C3b	+	70% +	
GE4	−	90% +	stable triploid karyotype, 59–60,XXY [cp12]
A3-B6	−	95% +	
GD2	−		
CC3	+		basically female mouse karyotype, though with an elevated level of tetraploidy and a population with only one X chromosome
CC2	+		The majority of cells were near tetraploid female with clonal aneuploidies

* HA4 and HE5 clones were used for blastocyst injection.

## Discussion

Studies determining the minimal amount of dystrophin and the minimal number of skeletal fibers that need to express dystrophin in the skeletal muscle to correct or ameliorate disease have been performed previously [3], [4], [27]. Lack of pathology has been described in mosaic mice with roughly 50% of the fibers expressing dystrophin [3], [27]. Hearts from aging mdx heterozygous mice, which express dystrophin in 50% of the cardiomyocytes, unlike those from mdx mice, do not develop cardiomyopathy [40]. Introduction of transgenic dystrophin in mdx mice corrects disease when the muscle expresses more than 20% of the total dystrophin protein [3]. It appears therefore that only a fraction of the numbers of fibers and of the total amount of dystrophin is critical for disease correction.

Previously we described that WT ESCs rescue the lethality of Id knockout embryos, a mouse model of the thin myocardial syndrome, and that ESC-derived secretion factors accounted for the reversion of the phenotypes in the embryonic heart and brain [41]–[43]. Here we show that although dystrophin is a key structural protein, low numbers of WT ESCs (10–30% chimerism) incorporated into mdx mice prevent disease at the morphological and functional level. Surprisingly, rather than containing 10–30% of dystrophin levels, we found that the mosaic muscle contained higher levels of dystrophin, in some cases comparable to wild-type. We also found a tight correlation between levels of dystrophin (western blot) and percentage of fibers expressing dystrophin (immunofluorescence) or β-galactosidase (Xgal staining). Limb (quadriceps) and thoracic (pectoralis) muscles exhibited the highest levels of dystrophin protein and the highest levels of Xgal staining, while the diaphragm had lower levels of dystrophin protein and lower levels of Xgal staining (but higher levels than other tissues that do not syncitialize, like heart). Despite differences in levels, all muscles with 10–30% of incorporation showed improvement of pathology. We determined that β-gal expansion in the skeletal muscle does not require the presence of an mdx component, as is also observed in WT/WT chimeras. Likewise, β-gal expansion occurs during development, since we have observed this phenomenon in the musculature before birth. β-gal expansion correlates with multinucleation, as it is observed only at late stages of development (when multinucleation is apparent) but not at midgestation (when multinucleation is not apparent). Despite β-gal expansion and an increase in dystrophin protein in the mosaic muscle, the degree of chimerism is low, as it contains only one-fifth of WT ESC-derived myonuclei. Since the degree of chimerism in these muscles is low the net increase in dystrophin production may be due to enhanced production, and/or perhaps, decreased destruction. The effect of overproduction is not because the WT component is derived from ESCs, as mdx+/− skeletal muscle (which does not contain ESC-derived myonuclei) also shows WT levels of dystrophin (despite that 50% of the myonuclei expresses dystrophin). However, this effect of overproduction and spatial expansion is specific for the multinucleated skeletal muscle, as neither heterozygous nor chimeric hearts overproduce dystrophin. Skeletal muscle has the distinct property to regenerate via activation of satellite cells. In this regard, we have observed that blastocyst injection of ESCs also results in mosaicism of the myogenic cell population, and that this population is capable of producing Xgal posititve, multinucleated myotubes. Thus, the chimeric pool of myogenic cells could be responsible for regenerating the muscle with new fibers containing ESC-derived myonuclei, and therefore for providing a continuous supply of dystrophin. The rescue by WT ESCs is dose-dependent, as WT/mdx mice containing less than 5% of WT chimerism do not exhibit improvement of pathology or function. Patches in the skeletal muscle of 10–30% WT/mdx chimeras contained little dystrophin. However, even these areas displayed an improvement of the pathology. Dystrobrevin, a member of the DGC [18], [20], which is a dystrophin-related protein, is stabilized in the fibers containing minimal dystrophin, but fibers devoid (with undetectable levels) of dystrophin do not display dystrobrevin. Thus, a minimal amount of dystrophin is required for co-localization of dystrobrevin to the sarcolemma. Utrophin, another dystrophin-related protein, is present in WT/mdx fibers with negligible dystrophin, but unlike dystrobrevin, utrophin is normally present in the sarcolemma of the mdx fibers. Little regeneration occurs in these fibers, suggesting that there is little degeneration and that the DGC is assembled and functional. Dystrobrevin can potentially replace some functions of dystrophin especially those associated with the binding of syntrophin and through it to nNOS [16], [44], but recent experiments show that forced expression of a functional dystrobrevin isoform to the sarcolemma does not ameliorate disease in the mdx mice [16]. Thus, a minimal presence of dystrophin (as observed in the low dystrophin fibers of the WT/mdx chimeras) appears to be critical to improve the pathology.

The muscle is not the sole target of the ESC-derived dystrophin, as the chimeras also display increased fat mass. The increase in cell mass is not due to an increase in cell size, as the chimeric adipocyte size is not enlarged. Unlike the muscle, the adipose tissue does not express full-length dystrophin. Although the fat does not express dystrophin, the rescue depends on dystrophin, as mdx ESCs without dystrophin fail to revert the fat phenotype. This suggests that there are dystrophin-expressing tissues involved in the rescue of the mass of the adipose tissue, and supports the notion of mechanisms of communication between tissues, in this case fat and non-fat tissues. It is possible that the primary dystrophin-positive corrective source is the chimeric skeletal muscle, and that upon normalization of the muscle phenotype, other tissues like the fat are normalized. It is also possible that other dystrophin-positive tissues (e.g. neuronal, smooth muscle, heart) play corrective roles. But what could be the reciprocal effect of the recovered chimeric fat mass on the state of the rescued muscle? The fat tissue is a rich source of circulating bioactive factors that may act in a paracrine manner [45]. In a dystrophic muscle, the adipose tissue replaces necrotic areas [46]. Thus, fat and muscle are intimately related. Indeed, brown fat cells arise from precursors that express Myf5, a gene previously thought to be expressed only in the myogenic lineage, and muscle/fat inter-conversion can be bi-direcionally achieved by manipulating key switch transcriptional regulators [47]. In this regard, we noticed that a set of muscle contractile proteins (cardiac and skeletal myosin, tropomyosin and troponin isoforms) is differentially upregulated in the mdx fat (relative to the WT fat). This enriched pattern of “muscularized” fat is also apparent in the chimeric fat. Along with changes in contractile markers, a set of growth/hypertrophic factors is altered in the chimeric fat. For example, follistatin-like is upregulated in both the mdx and chimeric fat (relative to WT). Follistatin and follistatin-like are circulating factors that bind and antagonize myostatin, promoting hypertrophy in the skeletal muscle [39], [48], [49], and the muscle of the WT/mdx chimeric mice shows signs of hypertrophy (our study). In addition to follistatin-like, other families of secreted factors are altered in the chimeric fat, like insulin-like growth factor (IGF), its binding proteins (IGFBP) and Wnts (unpublished data). Thus, the presence of increased adipose mass coupled with the presence of upregulated key growth/hypertrophic factors observed in the chimeric fat makes this tissue a candidate source of bioactive signals. These paracrine signals may have an impact on the growth/hypertrophic state of a muscle that has only a fraction of dystrophin, like WT/mdx muscle. Whether the changes observed in the chimeric fat play a role in the corrections of the chimeric musculature, particularly in the fibers that have negligible levels of dystrophin, it remains to be determined. It is noteworthy to point out that DMD patients undergo a biphasic mode of fat gain/loss with an increase in fat mass in the prepubertal age followed by a decline in fat mass after 13 years of age. It is important for such patients to maintain their body weight to prevent the deterioration of respiratory function and to prolong their life [50]. Importantly, our results suggest that ESC-derived dystrophin is required for the global corrections observed in the chimeric mice.

## Materials and Methods

### Embryonic stem cells

WT *LacZ*-marked R26 embryonic stem cells were developed and provided by Phillipe Soriano. WT R26 cells grow in DMEM with high glucose, 15% FCS, glutamine, nonessential aminoacids, β-mercaptoethanol, antibiotics and on SNLa76/7 STO cells, which constitutively express LIF.

### Mouse colonies

Hemizygous C57BL/10ScSn-*mdx*/J (mdx for muscular dystrophy gene localized to the X-chromosome) males and homozygous C57BL/10ScSn-*mdx*/J (mdx) females were purchased from Jackson lab. The colony was maintained by crossing mdx males with mdx females. To generate mdx+/− females, WT males (C57BL/10) were crossed with mdx females. Male WT or mdx mice, and female mdx+/− mice were analyzed. All animal experiments were approved by the Institutional Animal Care and Use Committee (IACUC) of the University of Medicine and Dentistry of New Jersey.

### Establishment of mdx, LacZ-marked ESCs

Hybrid mdx/Y∶R26/+ males were generated by mating R26 males with C57BL/10ScSn-*mdx*/J females and selecting for progeny carrying both the R26 allele and mdx mutation. Four weeks old C57BL/10ScSn-*mdx*/J females purchased from Jax labs were superovulated (12 females) and mated with mdx/Y∶R26/+ hybrid males. Mated females were checked for plugs the next morning to identify successfully mated animals.

ES cells were established using a modified version of the method described by Andras Nagy et al. (Manipulating the Mouse Embryo, 3rd ed, CSHL Press). Plugged females were euthanized 3 days after mating and uterine horns were flushed with M2 medium to retrieve blastocysts as well as late morula stage embryos. Flushed embryos were maintained at 37°C in 5% CO2 in micro-drops of M16 medium under oil for 2 days to allow hatching from the zona. After this incubation, hatched blastocysts were transferred individually into wells of a 96-well plate, containing mouse embryo fibroblasts (MEF) as feeders and cultured in ESC medium (DMEM with high glucose, supplemented with 15% FCS, 0.1 mM β-mercaptoethanol, 4 mM L-glutamine, 1× Non-essential amino acids and 1000 U/ml LIF) until outgrowths of the inner cell mass (ICM) are evident. Healthy ICM outgrowths were disaggregated between day 7 and 8, and plated onto fresh feeder cells. Cultures were monitored for ES cell colonies and all healthy colonies were passaged and expanded to establish ES lines. A total of 20 ES cell lines were obtained. Of these, thirteen were analyzed for AP and lacZ activities and their karyotype examined.

### Analysis of the established ES cell lines

#### Alkaline Phosphatase (AP) activity

The ES clones were cultured on 24-well gelatin-coated dishes without feeders until confluency. Cells were rinsed twice with PBS, fixed with 4% para-formaldehyde at room temperature for 15 minutes, and then rinsed with PBS. Fixed cell samples were tested for AP activity by incubating with substrate (5 mg Sodium alpha-naphthyl phosphate, 5 ml 5% borax, 44.6 ml H2O, 0.3 ml 10% MgCl2, 12 mg Fast Red TR, PH to 9.2) at 37°C for 30 minutes. After incubation, cells were rinsed and examined for AP staining.

#### Karyotype Analysis

The ES clones were cultured in 6-well plates coated with gelatin without feeders and grown to about 70% confluency. Cells were then karyotyped by the MSKCC Molecular Cytogenetics Core Facility.

#### lacZ Analysis

To verify that the mdx ES clones contained the R26 β-gal gene trap allele, ES cells were grown in gelatin-coated 24-well plates until confluency. Cells were rinsed twice with PBS, then once with buffer A (10 mM Na-Phosphate, PH 7, 150 mM NaCl, 1 mM MgCl2). Samples were fixed with 1% glutaraldehyde in buffer A for 5 minutes at room temperature. After fixing, cells were rinsed with buffer B (3.3 mM K3Fe(CN)6, 3.3 mM K4Fe(CN)6•3H2O in buffer A), and then tested for β-gal activity by incubating with the substrate X-gal (0.3% X-Gal in buffer B) at 37°C in dark for 3 hours. Samples were rinsed and either examined immediately for stained cells or kept in PBS at 4°C, in the dark, until ready for analysis.

### Generation of chimeric mice

3-week old mdx (C57BL/10ScSn-*Dmd^mdx^*/J, Jax labs) females were superovulated and mated with mdx males (Jax labs). Blastocysts were collected at 3.5 days afer mating, injected with either 15 WT R26 or 15 mdx R26 ES cells. Injected blastocysts were then transferred into the uteri of pseudopregnant females and allowed to develop to term. A combination of X-gal staining/eosin counterstaining on tail tip cryosections at 1 week of age, on liver, heart or lung cryosections at 4 months of age, semi quantitative PCR of the LacZ transgene (liver, muscle) and dystrophin immunofluorescence on heart cryosections (in case of WT/mdx chimeras at 4 months of age was used to characterize percentage of ESC incorporation. All chimeric animals analyzed were males.

### Immunofluorescence (IMF), X-gal histochemistry, western blot (WB)

Diaphragms, pectoralis, quadriceps and hearts were collected at 4 months of age, OCT embedded and cryosectioned (12 µm). Antibodies reactive with dystrophin (dys2 for IMF and dys1 for WB, Novocastra), utrophin (generously provided by Dr. Fritschy, University of Zurich), dystrobrevin-α (610766, BD Transduction Laboratories), syntrophin (MA1-745, ABR), neuronal nitric oxide synthase (PA1-038, ABR), sarcoglycan β (Novocastra), embryonic myosin heavy chain F1.652 (Developmental Studies Hybridoma Bank, DSHB) slow myosin heavy chain (MHCs, Novocastra) were used. Before OCT embedding, hearts were placed in Krebs-Ringer solution to allow arrest in diastole. X-gal staining was performed on sections with X-gal (1 mg/ml) in PBS buffer containing 5 mM potassium ferricyanide, 5 mM potassium ferrocyanide and 2 mM MgCl2 overnight at 37°C. Embryos were either whole mount X-gal stained and subsequently cryosectioned (E11.5) or cryosectioned first and subsequently X-gal stained (E17.5). Myogenin F5D (Developmental Studies Hybridoma Bank, DSHB) or Myosin Heavy Chain MF20 (DSHB) antibodies were used to detect myogenic areas in E11.5 or E17.5 respectively. In the case of whole mount staining at E11.5, the X-gal wash buffer contained 0.02% Nonidet P-40. X-gal stained sections were eosin counterstained before mounting. For western blot, a 4–20% gradient acrylamide gel was used. High molecular weight protein standards (HiMark, Invitrogen and Precision Plus, Biolab) were used. The intensities of the WB exposures were quantified using Quantity One software on a GS800-Densitometer (Bio-Rad). The relative expression levels for the dystrophin bands were normalized using tubulin bands within the same linear range of detection. The muscle area collected for WB was not previously stained (Xgal or dystrophin IMF), and since the ESCs distribute non-uniformly in the muscle with intermingled areas of high and low dystrophin, the protein extracts contained varying combinations of both high and low dystrohin components. Because there were more areas with high dystrophin than with low dystrophin in most of the 10–30% muscles (i.e. pectoralis and quadriceps), the values obtained in the WB were higher than 60% (see Table I).

### Percentage of dystrophin-positive fibers (or cardiomyocytes) and of X-gal positive fibers (or cells)

The number of dystrophin- and utrophin-positive cardiomyocytes (or skeletal muscle fibers) was counted under a fluorescent microscope (200×, 20 fields). To calculate the percentage of dystrophin-positive cells (or fibers), the number of dystrophin-positive cells (or fibers) was divided by the total number of cells (or fibers) (dystrophin- plus utrophin-positive cells or fibers). Very rarely a fiber contained dystrophin and utrophin staining, and it was counted as dystrophin-positive if it contained more than 50% of sarcolemma staining. The number of X-gal positive cells (or fibers) and eosin positive liver, lung or cardiac cells (or skeletal fibers) was counted under a bright microscope (200×, 20 fields). In most cases the fibers stained dark blue, but in some cases fibers were light blue. Dark and light blue fibers were counted as X-gal positive. To calculate the percentage of X-gal positive cells (or fibers), the number of X-gal positive cells (or fibers) was divided by the total number of cells (or fibers) (X-gal positive plus eosin positive cells or fibers). X-gal stained sections were also counterstained with light hematoxylin (for nuclei visualization) when eosin alone did not demarcate cell limits in the liver, heart or in the lung.

### Semi quantitative genomic PCR

Genomic DNA from 4 chimeras was isolated from LacZ+ R26 WT/mdx, LacZ+ R26 mouse and LacZ- WT mouse skeletal mouse tissue (quadriceps) by overnight digestion in SDS/Proteinase K buffer followed by phenol/chloroform extraction. 50 ng of genomic DNA were used in PCR with Jackson Labs' primers for genotyping the LacZ transgene incorporated in the R26 locus (Jackson Labs IMR0092-AATCCATCTTGTTCAATGGCCGATC&IMR3449-CCGGATTGATGGTAGTGGTC). 2 ng of the same DNA were used in PCR with G3PDH primers (Clontech) for internal control. PCR was performed with puReTaq Ready-To-Go PCR beads (GE Healthcare) for 31 and 35 cycles in the log phase. Scans were performed on Typhoon 8600 Variable Mode Imager (Molecular Dynamics), and quantitation done using ImageQuant.

### Functional analysis

Mice aged ∼8 weeks with 10–30% (n = 2) and <5% (n = 2) of ESC chimerism and WT (n = 4) and mdx (n = 3) controls were deeply anesthetized (2 mg xylazine-20 mg ketamine/100 g body mass ip). Both fast-twitch extensor digitorum longus (EDL) muscles were excised and incubated at 30°C in an oxygenated (95% O_2_-5% CO_2_) physiological salt solution (PSS; pH 7.6) containing (in mM) 120.5 NaCl, 4.8 KCl, 1.2 MgSO_4_, 20.4 NaHCO_3_, 1.6 CaCl_2_, 1.2 NaH_2_PO_4_, 10.0 dextrose, and 1.0 pyruvate. Silk suture (4-O) was tied to the distal and proximal tendons of the EDL at the myotendinous junctions. Muscles were then fixed between a clamp at the base of the bath and arm of a dual-mode servomotor system (300B, Aurora Scientific), or an isometric force transducer (Grass FT03), at a resting tension (L_0_) of 1.0 g. EDL muscles were maintained at L_0_ by a stepper motor [51]. The servomotor arm, stepper motor and electrical stimulator were controlled by Dynamic Muscle Control software (DMC Version 4.1.6, Aurora Scientific) to obtain isometric force data. Isometric twitch and tetanic data were obtained. Length of the muscles was obtained with a micrometer; each muscle was weighed to the nearest 0.1 mg using an A-200D electronic analytical balance (Denver Instruments, Denver, Colorado) and then muscles were snap frozen in liquid nitrogen for subsequent sectioning and WB analysis. Muscle cross-sectional area (CSA) was determined as previously described [52]. Twitch and tetanic forces were normalized to muscle CSA to obtain twitch and tetanic stress (g/mm^2^). As every muscle has a distinct pattern of ESC incorporation in a chimera, the values obtained from the 2 EDL muscles from the same animal (2 mice) were independently considered, and thus, n = 4 per chimeric category (10–30% and <5%). The degree of chimerism was estimated by Xgal staining of sections from tail biopsies, and further confirmed upon sacrifice by Xgal and dystrophin (heart) and Xgal (liver) staining. Dystrophin presence in the EDL muscles was confirmed by IMF and by WB.

### Cross-sectional area measurements

Digitized images of diaphragm sections examined were acquired using an upright microscope with camera driven by diagnostic software. Image files were analyzed using ImageJ software (NIH). Muscle fibers in cross-section were traced onscreen using a mouse-driven cursor, and the dimensions of highlighted fibers calculated within the software package based on a calibrated screen pixel-to-actual size ratio [53].

### Primary cell cultures

Myogenic cultures were prepared from R26 WT/mdx mice according to an established protocol [54]. Cells were isolated from diaphragm and quadriceps muscles after careful dissection of the muscles to minimize connective tissue contribution. Collected muscles, processed separately by source muscle, were enzymatically digested sequentially by collagenase-1, dispase and trypsin. In parallel, non-digested muscles were minced and cultured. Released single cells (or minced explants) were cultured in gelatin-coated plates in a serum-rich growth medium consisting of DMEM supplemented with 25% fetal bovine serum (Hyclone, Logan, UT), 10% horse serum (Hyclone), 1% chicken embryo extract and antibiotics. Cells were allowed to expand for up to two weeks and subjected to differentiation into contracting myotubes for 5 days in the presence of DMEM supplemented with 0.5% horse serum, Myogenic cultures containing mononuclear cells and/or myotubes were subsequently Xgal stained and photographed.

### Microarray analysis

Total RNA from pectoralis muscle was isolated from a 4-month old WT, mdx, WT/mdx or mdx/mdx mice (RNeasy, QIAGEN). RNAwas converted to cDNA, cRNA, and hybridized to DNA sequences contained in the MOEA2.0 (14,000 murine well-characterized genes, Affymetrix) chip. Information from at least duplicate samples was compared and filtered by fold change>2 (mdx vs WT and WT/mdx vs WT) or by fold change>1.5 (mdx/mdx vs WT) and statistical p-value<0.001. Data sets have been deposited in Gene Expression Ommibus (GEO), with accession number GSE12580.

### Data analysis

Results are presented as mean±s.e.m or as a range. Statistical comparison was performed with nonparametric two-tailed unpaired analysis of variance. A probability value of <0.05 was considered to be statistically significant.

## Supporting Information

Table S1 Comparison of gene expression profiles between 4 month-old mdx and WT pectoralis muscles
(0.45 MB XLS)

Table S2 Comparison of gene expression profiles between 4 month-old WT/mdx (20% of chimerism) and WT pectoralis muscles
(0.33 MB XLS)

Table S3 Comparison of gene expression profiles between 4 month-old mdx and WT adipose tissues
(1.85 MB XLS)

Table S4 Comparison of gene expression profiles between 4 month-old WT/mdx (20% of chimerism) and WT adipose tissues
(2.05 MB XLS)

Table S5 Comparison of gene expression profiles between 4 month-old mdx/mdx (20% of chimerism) and WT pectoralis muscles
(1.23 MB XLS)
